# Data on the relationship between traveller perceived value and traveller intention to revisit a destination

**DOI:** 10.1016/j.dib.2019.104435

**Published:** 2019-08-27

**Authors:** Tinashe Chuchu

**Affiliations:** University of Pretoria, South Africa

**Keywords:** Affective, Cognitive, Destination image, Traveller, Tourists

## Abstract

Data was collected at the OR Tambo Airport in Johannesburg South Africa from 503 willing international tourists. The survey was self-administered over a two-month period. Due to the absence of a sampling frame, non-probability sampling was adopted in selecting participants. A unique conceptual model was developed to test the causal effect of traveller perceived value on cognitive and affective destination image as well as on traveller intention to revisit. In addition, the direct effect of cognitive and affective destination image on traveller intention to revisit was also measured. Analysis of data involved descriptive statistics and structural equation modeling conducted in the Statistical Package for the Social Sciences (SPSS) 25 and Analysis of Moment Structures (AMOS) 25 respectively. Descriptive statistics produced frequencies on gender, age, travels, purpose of trip and holidays associated with each respondent. Structural equation modeling was conducted following a two-step process. First, confirmatory factor analysis followed by hypothesis testing. Further research could assess the possibility of a link between affective and cognitive destination image.

**Table:** Specifications Table

Subject	Tourism, Leisure and Hospitality Management
Specific subject area	Consumer behaviour, Marketing
Type of data	Table Figure
How data were acquired	Data was acquired through a self-administered survey at the OR Tambo International Airport in Johannesburg, South Africa from willing international tourists.
Data format	Raw, analysed and statistical data
Parameters for data collection	To qualify for inclusion in the sample the participant had to be identified as an international tourist (a non-resident of South Africa).
Description of data collection	Trained field workers distributed surveys to willing international tourists inside the OR Tambo International airport. All surveys were hard copy print-outs.
Data source location	Johannesburg, South Africa 26.1367° S, 28.2411° E
Data accessibility	Data is included in this article
Related research article	Tinashe Chuchu Destination Marketing: A Study into International Airport Service Experience, Destination Image and Intention to Revisit South Africa University of the Witwatersrand Wired Space Repository DOI: 10.13140/RG.2.2.11768.70402

**Table:** 

**Value of the data** • The data helps explain the relationship between travellers' perception of value, views and thoughts of a destination and whether all this would influence them to revisit the place? • Marketers, tourism practitioners such as tour operators, researchers on destination marketing and policy makers stand to benefit from these data • These data could be used to test the mediation effect of affective and cognitive destination image between traveller perceived value and traveller intention to revisit given that a direct relationship between the two does not exist. Alternatively, these data could be used for regression analysis to see if traveller perceived value, cognitive and destination image are all direct antecedents of traveller intention to revisit. Lastly, these data could be used to measure the direct relationship between affective and cognitive destination image. • The additional value of this data is that it has a substantial sample size (503 respondents) and an extra two additional constructs were not measured SS (airport servicescape) and CNDI (conative destination image). Future researchers could use these data and incorporate these two variables in potential rival models and produce interesting findings.

## 1 Data

**Fig. 1:**
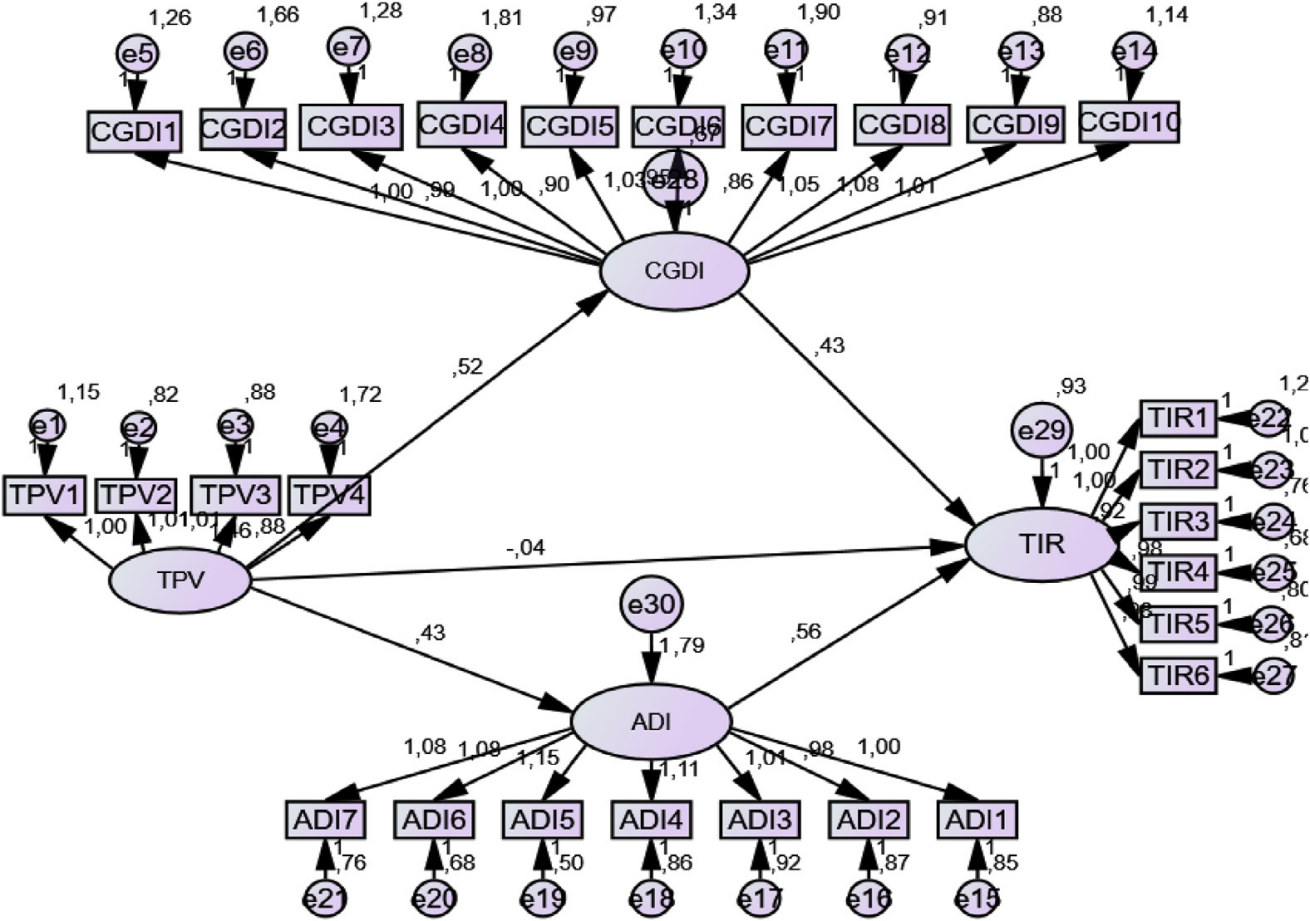
Structural Model. Key: TPV; Traveller perceived value, CGDI; Cognitive destination image, ADI; Affective destination image, TIR; Traveller intention to revisit.

The data is presented through four tables and one figure. Table 1 presents the sample profile showing demographic characteristics of the participants such as gender and age. Table 1 also shows the participants’ frequency of travels and the purposes of their trips as well as frequency of holidays. Table 2 presents the model fit criteria and the corresponding outcomes for each indicator. In Table 3, the accuracy analysis statistics are presented which include reliability and validity measures. Fig. 1, illustrates the structural model showing all the outcomes of the proposed hypotheses. Lastly, Table 4 presents the hypotheses results.

**Table 1:** Sample profile.

	Representation
**Gender**	
Male	58,4%
Female	39,0%
Prefer not to say	2,6%
Total	100,0%
**Age**	
18–19	6,6%
20–25	22,5%
26–35	32,4%
36+	37,8%
No response	0,8%
Total	100,0%
**Frequency of travels**	
Once a week	4,2%
Often a week	5,0%
More than once a month	23,3%
At least once a year	52,1%
Other	15,5%
Total	100,0%
**Purpose of trip**	
Leisure	35,2%
Business	33,4%
Educational purposes	16,5%
Medical reasons	2,2%
Other	12,7%
Total	100,0%
**Frequency of holidays**	
Every few years	14,7
Once every two years	5,6
Once a year	35,6
Twice a year	14,7
More than twice a year	19,7
Other	9,7
Total	100,0%

**Table 2:** Model fit.

Model fit criteria	CMIN//DF	GFI	CFI	IFI	NFI	RFI	TLI	RMSEA
Indicator value	2,531	0,907	0,948	0,949	0,918	0,901	0,937	0,055

CFA Model: Confirmatory factor analysis model; CMIN/DF: Chi-square; GFI: Goodness of fit index; NFI: Normed Fit index; RFI; Relative Fit Index; IFI: Incremental Fit Index; TLI: Tucker Lewis Index; CFI: Comparative Fit Index. RMSEA: Root Measure Standard Error Approximation.

**Table 3:** Accuracy analysis statistics.

Research Construct		Descriptive Statistics				Cronbach's Test		C.R. Value	AVE Value	Highest Shared Variance	Factor Loading
		Mean Value		Standard Deviation		Item-total	α value				
TPV	TPV1	4,648	4,721	1,617	1,593	0,692	0,833	0,833	0,560	0,245	0,741
	TPV2	4,761		1,520		0,705					0,828
	TPV3	4,853		1,543		0,689					0,812
	TPV4	4,620		1,692		0,575					0,587
CGDI	CGDI1	5,177	5,024	1,527	1,537	0,620	0,888	0,890	0,451	0,245	0,701
	CGDI2	4,748		1,645		0,600					0,629
	CGDI3	4,932		1,532		0,645					0,676
	CGDI4	4,630		1,639		0,567					0,556
	CGDI5	5,205		1,454		0,702					0,770
	CGDI6	4,899		1,521		0,590					0,631
	CGDI7	4,873		1,639		0,517					0,545
	CGDI8	5,368		1,450		0,684					0,740
	CGDI9	5,201		1,465		0,700					0,735
	CGDI10	5,209		1,496		0,645					0,693
ADI	ADI1	5,354	5,322	1,382	1,405	0,717	0,914	0,912	0,597	0,582	0,735
	ADI2	5,378		1,374		0,708					0,718
	ADI3	5,252		1,419		0,711					0,747
	ADI4	5,161		1,475		0,739					0,774
	ADI5	5,398		1,383		0,814					0,868
	ADI6	5,316		1,386		0,753					0,788
	ADI7	5,396		1,412		0,727					0,771
TIR	TIR1	5,091	5,127	1,723	1,594	0,745	0,917	0,918	0,652	0,621	0,781
	TIR2	5,056		1,675		0,775					0,785
	TIR3	5,408		1,489		0,761					0,838
	TIR4	5,175		1,524		0,777					0,898
	TIR5	4,940		1,579		0,773					0,778
	TIR6	5,089		1,575		0,772					0,758

Key: TPV; Traveller perceived value, CGDI; Cognitive destination image, ADI; Affective destination image, TIR; Traveller intention to revisit, CR: Composite reliability, AVE: Average variance extracted.

**Table 4:** Hypothesis results.

Hypothesis		Path coefficient (β)	P Value	Result
TPV ⇨ CGDI	(H_1_)	0.52	∗∗∗	Supported and significant
TPV⇨TIR	(H_2_)	−0.04	0,513	Not supported and insignificant
TPV ⇨ ADI	(H_3_)	0.43	∗∗∗	Supported and significant
CGDI ⇨ TIR	(H_4_)	0.43	∗∗∗	Supported and significant
ADI ⇨ TIR	(H_5_)	0.56	∗∗∗	Supported and significant

Key: TPV; Traveller perceived value, CGDI; Cognitive destination image, ADI; Affective destination image, TIR; Traveller intention to revisit, Significance level P < 0.01 (***).

## 2 Experimental design, materials, and methods

The research was quantitative in nature adopting the survey methodology. Due to the difficulty in obtaining a sampling frame of international tourists passing through the airport non-probability convenience sampling was adopted in appropriately selecting suitable participants. Questionnaire design was based on past research and adaptations were made where necessary.

## 3 Theoretical basis of proposed model

The study's structural model is presented in Fig. 1. Traveller perceived value and overall satisfaction are associated with the intention to revisit and recommend a destination [1]. Intentions to revisit a destination within the next 2 years can be predicted by satisfaction with one's last visit, perceived value of the last visit, and past behaviour Petrick et al. [2]. Perceived value mediates the relationship between destination image and revisit intention at the same time directly influencing revisit intention according to Cheng et al. [9]. Perceived Value has the potential to predict intentions to revisit [3]. Satisfaction is influenced by behavioral intention to revisit a destination, Kim et al. [4].

## 4 Structural equation modeling

Structural equation modeling was conducted using the two-step procedure proposed by [5], which assesses model fit comprising of confirmatory factor analysis (CFA) and hypotheses testing. Confirmatory factor analysis (CFA) was primarily performed to examine scale accuracy of the multiple-item construct measures using AMOS 25. Reliability checks were conducted in SPSS 25 in order to generate the Cronbach's alpha (α), item totals, means and standard deviations. Table 2 below shows the model fit criteria used for the study as well as indicator values for each criteria.

The measurement model produced a ratio of chi-squared value over degree-of-freedom of 2.531 which is acceptable as it falls below the 3, recommended by [6]. Other model fit indices that included the GFI, CFI, IFI, NFI, RFI and TLI were 0,907, 0,948, 0,949, 0,918, 0,901 and 0,937 respectively. All these model fit measures were above the recommended threshold of 0.9. The RMSEA was 0.055, which fell below the threshold of 0.08, recommended by Hooper et al. [7]. The accuracy analysis statistics are presented in Table 3.

Table 3 above indicates that most of means ranged from 4, 721 to 5, 322, while all Cronbach's alpha values were above the required 0.7. The standard deviation values were between 1 and 2 while all item totals were above 0.5. In addition, most CR values were above the recommended 0.6 while most of the AVE values were above the accepted level of 0.5. The AVE value of (TPV) is 0,560 which is greater that the square of the shared variance of (TPV) and (CGDI) which [(0,495) ^2^] = 0,245. This therefore proves the existence of discriminate validity, [8]. Composite reliability (CR) values and average variance extracted (AVE) values for each construct were generated using the following the formulae:

CRη= (Σλyi)2/[(Σλyi)2+(Σεi)]

Where

CRη = Composite reliability, (Σλyi) 2 = Square of the summation of the factor loadings; (Σεi) = Summation of error variances.

Vη = Σλyi2/ (Σλyi2+Σεi)

Where

Vη = Average Variance Extracted (AVE); Σλyi2 = Summation of the squared of factor loadings; Σεi = Summation of error variances”.

Table 4 presents results of hypothesis testing. H1 (Traveller perceived value and cognitive destination image, was supported and significant at p < 0.01 having (β = 0.52). H2 (Traveller perceived value and traveller intention to revisit), was not supported and insignificant at (β = −0.04). H3 (Traveller perceived value and affective destination image), was also supported at (β = 0.43). Lastly, H4 and H5 indicated that traveller perceived value is related to both affective and cognitive destination image at (β = 0.43) and (β = 0.56) respectively.

## 5 Ethical considerations

All surveys were anonymous. Permission to collect data on site at the OR Tambo International airport was granted by Airports Company South Africa while ethics clearance to conduct the research was awarded by the University of the Witwatersrand, Johannesburg.
